# Are the Pediatric Index of Mortality 2 and 3 equal predictors of mortality? An intensive care unit-based concordance study

**DOI:** 10.5935/0103-507X.20200096

**Published:** 2020

**Authors:** Daniela Patino-Hernandez, Alba Deyanira Quiñonez López, César Augusto Zuluaga, Ángel Alberto García, Oscar Mauricio Muñoz-Velandia

**Affiliations:** 1 Departamento de Medicina Interna, Hospital Universitario San Ignacio - Bogotá, Colômbia.; 2 Facultad de Medicina, Pontificia Universidad Javeriana - Bogotá, Colômbia.; 3 Departamento de Pediatría, Hospital Universitario San Ignacio - Bogotá, Colômbia.

**Keywords:** Pediatric Index of Mortality, Mortality, Infant, newborn, Cardiac surgery, Intensive care units, pediatric, Pediatric Index of Mortality, Mortalidade, Recém-nascido, Cirurgia torácica, Unidades de terapia intensiva pediátrica

## Abstract

**Objective:**

To determine the concordance of mortality risk classification through the use of the Pediatric Index of Mortality (PIM) 2 and 3.

**Methods:**

Through a retrospective cohort, we evaluated patients admitted to the pediatric intensive care unit between April 2016 and December 2018. We calculated the mortality risk with the PIM 2 and 3. Analyses were carried out to determine the concordance between the risk classification obtained with both scales using unweighted and linearly weighted kappa.

**Results:**

A total of 722 subjects were included, and 66.6% had a chronic condition. The overall mortality was 3.7%. The global kappa concordance coefficient for classifying patients according to risk with the PIM 2 and 3 was moderate at 0.48 (95%CI 0.43 - 0.53). After linear weighting, concordance was substantial at 0.64 (95%CI 0.59 - 0.69). For cardiac surgery patients, concordance for risk classification was fair at 0.30 (95%CI 0.21 - 0.39), and after linear weighting, concordance was only moderate at 0.49 (95%CI 0.39 - 0.59). The PIM 3 assigned a lower risk than the PIM 2 in 44.8% of patients in this subgroup.

**Conclusion:**

Our study proves that the PIM 2 and 3 are not clinically equivalent and should not be used interchangeably for quality evaluation across pediatric intensive care units. Validation studies must be performed before using the PIM 2 or PIM 3 in specific settings.

## Abstract (pt)

**Objetivo:**

Determinar a concordância da classificação do risco de mortalidade por meio do uso dos escores *Pediatric Index of Mortality* (PIM) 2 e 3.

**Métodos:**

Avaliação de uma coorte retrospectiva pela análise dos pacientes admitidos à unidade de terapia intensiva pediátrica entre abril de 2016 e dezembro de 2018. Calculamos o risco de mortalidade por meio do PIM 2 e do 3. Realizaram-se análises para determinar a concordância entre a classificação de risco obtida com ambas as escalas pela utilização do cálculo do Kappa não ponderado e linearmente ponderado.

**Resultados:**

Incluímos 722 pacientes, sendo que 66,6% destes tinham uma condição crônica. A mortalidade global foi de 3,7%. O coeficiente Kappa de concordância para classificação dos pacientes, segundo o risco com o PIM 2 e o 3, foi moderado: 0,48 (IC95% 0,43 - 0,53). Após ponderação linear, a concordância foi substancial: 0,64 (IC95% 0,59 - 0,69). Para pacientes de cirurgia cardíaca, a concordância para a classificação de risco foi regular: 0,30 (IC95% 0,21 - 0,39); após ponderação linear, a concordância foi apenas moderada: 0,49 (IC95% 0,39 - 0,59). O PIM 3 acusou um risco mais baixo do que o PIM 2 em 44,8% dos pacientes desse subgrupo.

**Conclusão:**

Nosso estudo comprova que o PIM 2 e o 3 não são clinicamente equivalentes e não devem ser usadas de forma intercambiável para avaliação da qualidade em diferentes unidades de terapia intensiva. Devem ser conduzidos estudos de validação antes que se utilizem os PIM 2 e 3 em situações específicas.

## INTRODUCTION

Multiple strategies have been developed to increase the rate of favorable outcomes of patients who require treatment in the pediatric intensive care unit (ICU).^(1)^ Among these strategies, models that predict the risk of mortality have become relevant for quality evaluation^(2)^ and as a means to objectively measure and compare processes over time,^(3)^ as they help to adjust for case-mix and severity of illness, allowing for comparisons among pediatric ICUs.^(2)^ One of the most frequently used scales is the Pediatric Index of Mortality (PIM), for which three versions have already been developed. The PIM 3 has been validated in different regions around the globe.^(4-6)^

The original PIM was released in 1997. The PIM calculates the risk of mortality based on eight variables that are routinely collected within the first hour of admission to the pediatric ICU.^(1,7)^ In 2003, a first update was developed by Slater et al.,^(1)^ yielding the PIM 2, which added three variables to the model. The new variables include changes in the main diagnosis on admission category (being admitted for postsurgical or postprocedural recovery, admission after extracorporeal circulation and low-risk diagnosis). Additionally, the specific diagnosis on admission is no longer used. Instead, the existence of a "high-risk" or "low-risk" diagnosis may be registered.

The predictive performance of the PIM 2 has been previously studied, and its discrimination and calibration vary significantly among populations.^(1,8,9)^ For instance, a study conducted in the United States found that its performance was poor in cardiac surgery patients.^(10)^ Additionally, a study conducted in Japan reported an overprediction of mortality in children over 12 months of age.^(4)^ Data from Latin America also revealed inadequate calibration in subgroup analysis, since mortality was higher than predicted in infants under 12 months, adolescents and during the postoperative period following noncardiac surgery.^(8)^ Some explanations for these variations include the sample size, diagnosis on admission, human resources and efficiency of healthcare systems.^(4,8)^ The aforementioned difficulties necessitated the second revision to this scale, which was published in 2013; this version was called the PIM 3, and it was developed using data from pediatric ICUs in three countries. The authors suggested that this score might be more representative of populations outside the original study group.^(11)^

The PIM 3 has 2 more variables than the PIM 2: postprocedural recovery, which is divided into three categories, and the "very high-risk diagnosis" variable, as well as other mathematical adjustments to physiological variables, such as systemic blood pressure, base deficit and the partial pressure of oxygen and fraction of inspired oxygen ratio (PaO_2_/FiO_2_), as described in table 1S (Supplementary material).^(1,11)^

Models that predict mortality risk have been used extensively in adult ICU settings^(10-13)^ and in pediatric settings.^(2)^^)^ However, when the scales are updated and some items vary, it is difficult to confidently state which version is better at predicting adverse outcomes.^(1,14)^ Even if the current version (PIM 3) is available free of cost and is easy to apply, no standardization exists in Colombia regarding which score should be used. Furthermore, some centers do not measure the risk of mortality as part of quality evaluation. Therefore, since the characteristics of intensive care units are variable in terms of complexity, diagnoses on admission, technological resources, and training of the human resources, it is essential to assess the degree of agreement among the available scores.

The concordance between the PIM 2 and 3 has not been assessed.^(15,16)^ This study aimed to assess the concordance of the classification of mortality risk obtained through the use of two scales (PIM 2 and 3).

## METHODS

We collected data from a retrospective cohort including all patients admitted to the pediatric ICU at a high complexity university hospital in Bogotá (Colombia) between April 1st, 2016, and December 31st, 2018. Our sample included patients with chronic medical conditions and patients during their postoperative period following elective or nonelective surgery or after cardiac bypass surgery. Patients who were transferred to other institutions in order to continue their treatment in critical care were excluded. The research and ethics committee at the *Hospital Universitario San Ignacio* and *Pontificia Universidad Javeriana* approved this research. The data were collected prospectively from electronic health records as the patients were admitted to the pediatric ICU. Two independent medical researchers who were not involved in patient care collected the variables of interest. If disagreements were found while classifying data, these disagreements were solved through consensus.

The following variables were collected from each subject: sex, days of pediatric ICU stay, age, diagnosis on admission, and diagnoses of any chronic conditions. Age was classified into the following categories: < 1 month, 1 - 11 months, 12 - 59 months, 60 - 119 months, and 120 - 215 months.^(1)^ Diagnoses on admission were adapted from the original PIM study,^(7)^ with a couple of modifications. For hematologic diseases, our hospital is a referral center for onco-hematologic diseases. For intoxication, there is a growing trend for admissions due to suicide attempts. As such, we aimed to include these particular traits in the study population. Variables were classified within the following categories: heart disease, trauma, hematological, neurological, respiratory, miscellaneous, postoperative noncardiac and intoxication. Variables were also classified by groups according to the presence of chronic disease: neurological, cardiovascular, respiratory, renal, gastrointestinal, hematological or immunological, metabolic, congenital defects, and malignancy. The operative definitions for variables included in the risk calculation are presented in table 1S (Supplementary material).

After the collection of the variables, we calculated the risk scores yielded by the PIM 2 and 3 for each of the participating subjects by using the equations described in the original studies.^(1,11)^ Risk categories were classified according to those reported in the original study,^(7)^ with slight modifications, from a low to a high risk of mortality as follows: 0 - 1%, 1.01 - 5%, 5.01 - 14%, 14.01 - 29%, and > 29%.

Then, to assess the concordance between the mortality risks predicted by the PIM 2 and 3, we performed a concordance analysis with the consistency approach, which considers the degree to which two or more tests coincide in a measurement in cases where a gold standard does not exist.^(17)^ We assessed the concordance among the predetermined risk groups by unweighted and linearly weighted kappa tests (Table 1) in order to differentially penalize the degree of agreement according to its magnitude^(17,18)^ for both the global sample and a subsample of cardiac surgery patients (defined as congenital cardiopathy or postoperative care for cardiac invasive procedures). This subgroup was analyzed due to previous reports of a poor performance of the scales in this group of patients.^(4,19)^ The strength of concordance was determined according to the Landis and Koch criteria: slight 0.01 - 0.20, fair 0.21 - 0.40, moderate 0.41 - 0.60, substantial 0.61 - 0.80; and almost perfect > 0.8.^(20)^ Data analysis was carried out using StataCorp 2015, Stata Statistical Software, Release 14 (StataCorp LP, College Station, TX).

**Table 1:** Linear weighting*

PIM 2/PIM 3	≤ 1%	1.01% - 5%	5.01% - 14%	14.01% - 29%	> 29%
≤ 1%	1	0.75	0.50	0.25	0
1.01% - 5%	0.75	1	0.75	0.50	0.25
5.01% - 14%	0.50	0.75	1	0.75	0.50
14.01% - 29%	0.25	0.50	0.75	1	0.75
> 29%	0	0.25	0.50	0.75	1

PIM - Pediatric Index of Mortality.

* Weight assigned to each degree of agreement.

## RESULTS

Our sample was composed of 722 subjects, among which the largest proportion (40.44%) was within the 1 to 11 months age category, and 66.62% of these subjects had a chronic condition. The most frequent diagnoses on admission were respiratory diseases, and a total of 37.40% subjects required mechanical ventilation. A total of 156 subjects (21.6%) were admitted under cardiac diagnoses, of which 39 (25%) were admitted for recovery after surgery with a bypass requirement (Table 2). The percentage of lost data was below < 5% for each variable.

**Table 2:** Description of the sample

Variables	
Age (months)	
1 - 11	292 (40.4)
12 - 59	205 (28.4)
60 - 119	92 (12.7)
120 - 215	133 (18.4)
Sex male	419 (58.0)
Pediatric ICU length of stay* (days)	
≤ 3 (standard)	286 (38.3)
4-14 (medium)	335 (44.9)
≥ 15 (prolonged)	125 (16.8)
Chronic diseases	481 (66.6)
Cardiovascular	132 (18.3)
Gastrointestinal	34 (4.7)
Genetic	53 (7.3)
Hematologic	28 (3.9)
Metabolic	11 (1.5)
Neurological	43 (5.9)
Renal	26 (3.6)
Respiratory	117 (16.2)
Malignant neoplasms	37 (5.1)
Diagnosis on admission	
Congenital heart disease	116 (16.1)
Noncardiac surgery recovery	94 (13.0)
Hematologic	42 (5.8)
Intoxications	21 (2.9)
Neurologic	34 (4.7)
Respiratory	290 (40.2)
Trauma	13 (1.8)
Miscellaneous	111 (15.4)
Mechanical ventilation	270 (37.4)
Days of mechanical ventilation	
≤ 3 (standard)	104 (13.9)
4 - 8 (medium)	103 (13.8)
> 8 (prolonged)	539 (72.3)
Cardiac bypass-requiring procedures	39 (5.4)

ICU - intensive care unit. Results expressed as n (%).

* Source: Pagowska-Klimek I, Pychynska-Pokorska M, Krajewski W, Moll JJ. Predictors of long intensive care unit stay following cardiac surgery in children. Eur J Cardiothorac Surg. 2011;40(1):179-84.^(21)^

Global mortality was 3.7%, which corresponded to 27 patients. Most of the evaluated subjects were classified according to the risk of mortality within the < 1% category when using the PIM 2 (45.7%), while the largest proportion of subjects was classified within the 1 - 5% category when using the PIM 3 (Table 3).

**Table 3:** Concordance between global risk classification according to Pediatric Index of Mortality 2 and 3

PIM 2	PIM 3					
	0% - 1%	1,01% - 5%	5,01% - 14%	14,01% - 29%	> 29%	Total
0% - 1%	237 (32.83)	92 (12.74)	1 (0.14)	0	0	330 (45.7)
1,01% - 5%	39 (5.40)	163 (22.58)	19 (2.63)	0	0	221 (30.61)
5.01% - 14%	2 (0.28)	36 (4.99)	38 (5.26)	2 (0.28)	0	78 (10.80)
14.01% - 29%	3 (0.42)	8 (1.11)	30 (4.12)	20 (2.77)	0	61 (8.45)
> 29%	0	0	10 (1.39)	11 (1.52)	11 (1.52)	32 (4.43)
Total	281 (38.92)	299 (41.41)	98 (13.57)	33 (4.57)	11 (1.52)	722 (100.0)

PIM - Pediatric Index of Mortality. Kappa 0.48 (95% confidence interval 0.43 - 0.53): moderate concordance. After linear weighting Kappa 0.64 (95% confidence interval 0.59 - 0.69): substantial concordance. Results expressed as n (%).

The mortality predicted by the PIM 2 was 6%, and the mortality predicted by the PIM 3 was 4%. Furthermore, the standardized mortality rate was 0.66 for the PIM 2 and 1.00 for the PIM 3. The global kappa concordance coefficient between the PIM 2 and 3 was moderate at 0.48 (95% confidence interval - 95%CI 0.43 - 0.53) (Table 3), and 252 patients (34.9% of the total) had discordant scores. The PIM 3 classified 114 patients (15.79%) within higher risk categories and 150 patients (20.78%) within lower risk categories than those classified by the PIM 2. After linear weighting (Table 1), the agreement according to the kappa coefficient was substantial at 0.64 (95%CI 0.59 - 0.69).

For the cardiac surgery patients, concordance for risk classification was fair at 0.30 (95%CI 0.21 - 0.39) (Table 4). The PIM 3 classified 9 patients (7.7%) within higher risk categories and 52 patients (44.8%) within lower risk categories than those classified by the PIM 2. Even after linear weighting, the concordance was only moderate at 0.49 (95%CI 0.39 - 0.59).

**Table 4:** Concordance between risk classification in cardiac surgery patients according to Pediatric Index of Mortality 2 and 3

PIM 2	PIM 3					
	0% - 1%	1.01% - 5%	5.01% - 14%	14.01% - 29%	> 29%	Total
0% - 1%	9 (7.76)	8 (6.90)	0	0	0	17 (14.70)
1.01% - 5%	0	37 (31.90)	1 (0.86)	0	0	38 (32.76)
5.01% - 14%	0	12 (10.34)	3 (2.59)	0	0	15 (12.93)
14.01% - 29%	0	7 (6.03)	16 (13.79)	3 (2.59)	0	26 (22.41)
> 29%	0	0	10 (8.62)	7 (6.03)	3 (2.59)	20 (17.24)
Total	9 (7.76)	64 (55.17)	30 (25.86)	10 (8.62)	3 (2.59)	116 (100)

PIM - Pediatric Index of Mortality. Kappa 0.30 (95% confidence interval 0.21 - 0.39): Fair concordance- After linear weighting Kappa 0.49 (95% confidence interval 0.39 - 0.59): moderate concordance. Results expressed as n (%).

## DISCUSSION

The PIM 2 and 3 have been used for quality assessment comparisons for some time, even though the concordance between them has not been evaluated. Our data suggest that the concordance between the PIM 2 and 3, as evaluators of mortality risk, is substantial after linear weighting. However, when considering the importance of correct risk assessment in pediatric populations, it would be desirable for the concordance to be almost perfect. Furthermore, the concordance is just moderate in cardiac surgery patients, with the PIM 3 assigning patients as having a lower risk than those classified through the PIM 2 in this subgroup.

In our sample, 66.2% of the patients had at least one coexisting chronic disease. This prevalence is relatively high when compared to previous studies, which ranged from 21% to 73.3%,^(22-24)^ and is related to the high complexity of the patients treated in our pediatric ICU. The most frequent diagnosis on admission was a respiratory disease, similar to that reported in previous studies conducted in Latin America, such as the study conducted by Arias Lopez et al. in which 36.2% of admissions were due to respiratory conditions.^(8)^ An interesting finding is that 21.6% of the sample was composed of cardiac surgery patients. This is a higher prevalence than that found in the study by Arias Lopez et al., allowing us to evaluate the concordance in this subgroup of patients.

Only fair concordance was found between the scales in the aforementioned subgroup,with substantial concordance being found only after linear weighting, suggesting that the mortality risk estimations obtained by the PIM 2 and 3 should not be interpreted as clinically equivalent. The greatest percentage of patients with a PIM 2 > 29% can be attributed to individuals diagnosed with cardiac disease (16% of the studied population), which has a greater weight in the PIM 2 than PIM 3. Furthermore, it is important to note that in our context, patients with cardiac conditions frequently had comorbidities, which was paired with difficulties in accessing healthcare services, and high prevalences of malnutrition and genetic diseases in this population may lead to worse outcomes. For patients with a mortality risk > 1%, we found that having a low-risk diagnosis apparently decreased the risk of mortality. In addition, the diagnosis "convulsive syndrome" was added to the PIM 3, yielding a lower risk in the PIM 2 compared to that in the PIM 3. This finding may explain why a higher proportion of patients may be classified as having a risk < 1% when assessed through the PIM 2 than through the PIM 3. This finding may underestimate the mortality risk in our population, particularly for patients admitted for bronchiolitis, which is also classified as a low-risk diagnosis but has been linked to poor outcomes in our country.^(25)^

In our study, the concordance between the PIM 2 and 3 was especially low among cardiac surgery patients. Limitations of the PIM in this subgroup of patients has been reported previously.^(1,11)^ These limitations may occur because the PIM do not evaluate the degree of complexity of cardiac surgeries. Other scores, such as the RACHS-1 (Risk Adjustment in Congenital Heart Surgery), may offer complementary information by recognizing individual risk according to each procedure's characteristics, but it does not assess the mortality risk. The ARISTOTLE complexity score aims to assess the potential for mortality, morbidity and technical difficulty. However, this score does not correspond to pediatric ICU settings.^(26,27)^ Finally, additional studies are necessary to assess the concordance between the PIM and Pediatric Index of Cardiac Surgical Intensive Care Mortality (PICSIM) scores, since the latter aims to assess mortality in cardiovascular surgery patients while combining the physiological, anatomical and procedural variables.^(28)^

Finally, we would like to add that our results demonstrate that the different scores cannot be considered to be equivalent for evaluation of the quality of care. Thus, the same scoring system must be used when comparing different pediatric ICU, or when comparing different time periods within a single pediatric ICU. Technological and scientific advances in ICU should be congruent with the development of tools for quality evaluation. Nonetheless, in developing countries, the latter may not always be applicable. As such, it may be valid to interpret the results after taking the conditions of specific pediatric ICU's into account, while bearing in mind that quality evaluation does require standardization regarding the use of a single score.

To the best of our knowledge, this is the first study to assess the concordance between risk scores obtained through the PIM 2 and 3. Another strength of our study is that the sample includes all patients admitted to the pediatric ICU within the study's predetermined timeframe. There are also some limitations to disclose. This is a concordance study, which means that we are able to assess equivalence but not to validate the risk scores for clinical use or quality evaluation. Future studies must address this knowledge gap. Furthermore, the retrospective collection of data may lead to bias related to incomplete or incorrectly reported information. However, the percentage of lost data was minimal, the imputation of data was performed according to the instructions disclosed in the original studies for both scales, and all the data were independently checked by two reviewers, reducing these risks.

However, since the application of the most up-to-date scale is not always possible, it is imperative to disclose the concordance among existing scores, taking into account that scales should undergo external validation to ensure they are useful in specific settings.^(29)^

## CONCLUSION

Our study proves that the PIM 2 and 3 are not clinically equivalent, particularly for cardiac surgery patients. These findings are important for clinicians in order to avoid using scoring systems interchangeably without considering the limitations of each. The standardized mortality ratio along with the length of stay have become important standards for quality evaluation among units.
