# Supplementary material for: Are the Pediatric Index of Mortality 2 and 3 equal predictors of mortality? An intensive care unit-based concordance study
Source: Rev Bras Ter Intensiva. 2020 Oct-Dec;32(4):578–84. doi: 10.5935/0103-507X.20200096 (PMC7853689; doi:10.5935/0103-507X.20200096)
Supplement: Supplementary file 1 [file rbti-32-04-0578-suppl01.pdf]

Are the Pediatric Index of Mortality 2 and 3 equal predictors of mortality? An intensive care unit-based concordance study

Pediatric Index of Mortality 2 e 3 são preditores de mortalidade iguais? Estudo de concordância com base em unidade de terapia intensiva

Daniela Patino-Hernandez[1,2], Alba Deyanira Quiñonez López[2,3], César Augusto Zuluaga[2,3], Ángel Alberto García[1,2], Oscar Mauricio Muñoz-Velandia[1,2]

Table 1S - Differences between the Pediatric Index of Mortality 2 and 3

The authors grouped diagnoses affecting risk of mortality into three categories: very high risk, high risk, and low risk
Patients with multiple weighted comorbidities were assigned to a categorical variable, and patients with multiple diagnoses were assigned to a single group
Regarding systemic blood pressure, an alternative to use of the variable "Blood pressure=120mmHg" being as a predictor was the use of third degree polynomials or quadratic polynomials, and in cases where the BP value was not found, the value 120 was used instead
Two transformations were considered for calculating base excess: the value representing absolute base excess and base excess as a quadratic function. When base excess was missing, a value of zero was used
Four approaches were considered for including the PaO_2 and the FiO_2 into the model. First, the ratio was calculated as follows: ($[FiO_2 \times 100] / PaO_2$). As in the PIM 2, the ratio was replaced with zero in cases where PaO_2 or FiO_2 were missing. Second, by replacing the ratio with 0.23 if the PaO_2 or FiO_2 were missing, as a derivative of the normal air PaO_2 value ($[0.21 \times 100] / 90$). Third, the natural logarithm of ($[PaO_2 / FiO_2] \times 100$) replaced the ratio by 430 if either PaO_2 or the FiO_2 were missing. Last, the absolute value for the difference between the calculated ratio ($[FiO_2 \times 100] / PaO_2$) and the normal value (0.23)
Finally, the following were established as categorical variables: postprocedural recovery ([0] no; [1] yes); recovery from a cardiac procedure requiring bypass ([2] yes); recovery from cardiovascular surgery without not requiring bypass ([3] yes) and recovery from noncardiac surgery, which displayed better operative characteristics for the model than binary variables of recovery and bypass-requiring procedures as described in the PIM 2

PaO_2 - partial pressure of oxygen; FiO_2 - fraction of inspired oxygen; PIM - Pediatric Index of Mortality.
